# Supplementary material for: Use of the “STANDARD G6PDTM” quantitative point-of-care test in neonates and infants
Source: PLoS One. 2026 Jul 10;21(7):e0346837. doi: 10.1371/journal.pone.0346837 (PMC13354085; doi:10.1371/journal.pone.0346837)

**Supplementary File 1**

Contents

[Tables 2](#_Toc230881652)

[**Table S1.** Mean (SD) haemoglobin levels by day of sampling and instrument 2](#_Toc230881653)

[**Table S2**. Intraclass coefficient of correlation for G6PD activity between Biosensor and Spectrophotometry 2](#_Toc230881654)

[**Table S3.** Mean differences haemoglobin (Biosensor – CBC) in g/dL by sampling day 2](#_Toc230881655)

[**Table S4.** Mean differences haemoglobin (Biosensor – Hemocue) in g/dL by sampling day 3](#_Toc230881656)

[**Table S5**. Mean (SD) values for selected blood indices over time 3](#_Toc230881657)

[**TableS6.** Summary of Biosensor data in neonates, infants, older children and adults. G6PD activity is expressed in U/gHb. 3](#_Toc230881658)

[Figures 4](#_Toc230881659)

[**Figure S1.** Age at sampling of the first capillary blood sample 4](#_Toc230881660)

[**Figure S2.** Individual participants’ G6PD activity over time 5](#_Toc230881661)

[**Figure S3.** Scatter plot of activity by Biosensor in cord blood against capillary sample (<24h of life) 6](#_Toc230881662)

[**Figure S4.** Scatter plot of activity by Biosensor in cord blood against capillary sample (day 7) 6](#_Toc230881663)

[**Figure S5**. Change over time by G6PD Status (Deficient-, Intermediate-, Normal-) in: A. Haemoglobin concentration, B. White blood cells, C. Red blood cells and D. Reticulocytes. 7](#_Toc230881664)

[**Figure S6**. Bland-Altman plot of G6PD results in **A**. cord blood, **B**. capillary blood collected within 24h of life, **C**. Day 7, **D**. Day 28 and **E**. Month 4. 8](#_Toc230881665)

[**Figure S7.** Bland-Altman plot for haemoglobin detected by Biosensor against CBC 9](#_Toc230881666)

[**Figure S8.** Bland-Altman plot for haemoglobin detected by Biosensor against Hemocue 9](#_Toc230881667)

# Tables

## **Table S1.** Mean (SD) haemoglobin levels by day of sampling and instrument

| Sampling day |  | Biosensor Hb (g/dL) | Complete blood count Hb (g/dL) | Hemocue (g/dL) |
| --- | --- | --- | --- | --- |
| CB | N | 74 | 74 | 73 |
|  | **Mean** | **16.0** | **14.6** | **15.0** |
|  | SD | 1.9 | 1.5 | 1.3 |
| <H24 | N | 74 | 75 | 73 |
|  | **Mean** | **19.9** | **17.9** | **18.7** |
|  | SD | 2.6 | 2.3 | 2.4 |
| D7 | N | 75 | 75 | 75 |
|  | **Mean** | **18.3** | **16.1** | **16.9** |
|  | SD | 2.2 | 1.9 | 1.8 |
| D28 | N | 75 | 75 | 75 |
|  | **Mean** | **14.4** | **12.4** | **12.9** |
|  | SD | 2.1 | 1.7 | 1.7 |
| M4 | N | 70 | 69 | 69 |
|  | **Mean** | **13.1** | **11.1** | **11.5** |
|  | SD | 1.7 | 1.0 | 0.9 |

## **Table S2**. Intraclass coefficient of correlation for G6PD activity between Biosensor and Spectrophotometry

| **Sampling day** | **N** | **ICC (95%CI)** | **P** |
| --- | --- | --- | --- |
| Overall | 367 | 0.944 (0.926-0.958) | <0.01 |
| CB | 74 | 0.925 (0.862-0.957) | <0.01 |
| <24H | 74 | 0.948 (0.919-0.967) | <0.01 |
| D7 | 75 | 0.950 (0.923-0.968) | <0.01 |
| D28 | 75 | 0.966 (0.945-0.979) | <0.01 |
| M4 | 69 | 0.926 (0.820-0.963) | <0.01 |

## **Table S3.** Mean differences haemoglobin (Biosensor – CBC) in g/dL by sampling day

|  | CB | <H24 | D7 | D28 | M4 | Overall |
| --- | --- | --- | --- | --- | --- | --- |
| N | 74 | 74 | 75 | 75 | 69 | 367 |
| Mean | 1.40 | 2.03 | 2.17 | 1.99 | 1.99 | 1.92 |
| SD | 1.77 | 1.48 | 1.39 | 1.22 | 1.41 | 1.48 |
| LoA | -2.07 to 4.88 | -0.88 to 4.94 | -0.56 to 4.90 | -0.40 to 4.38 | -0.78 to 4.76 | -0.99 to 4.82 |

## **Table S4.** Mean differences haemoglobin (Biosensor – Hemocue) in g/dL by sampling day

|  | CB | H24 | D7 | D28 | M4 | Overall |
| --- | --- | --- | --- | --- | --- | --- |
| N | 73 | 73 | 75 | 75 | 69 | 365 |
| Mean | 0.99 | 1.15 | 1.41 | 1.54 | 1.61 | 1.34 |
| SD | 1.71 | 1.53 | 1.23 | 1.15 | 1.41 | 1.43 |
| LoA | -2.36 to 4.34 | -1.86 to 4.15 | -1.00 to 3.82 | -0.71 to 3.79 | -1.16 to 4.38 | -1.46 to 4.14 |

## **Table S5**. Mean (SD) values for selected blood indices over time

|  | **CB** | **<24H** | **D7** | **D28** | **M4** |
| --- | --- | --- | --- | --- | --- |
| **WBC (10^3^/uL)** | 15.62 | 22.52 | 11.77 | 9.68 | 11.68 |
| *SD* | *4.62* | *5.63* | *2.27* | *2.14* | *3.36* |
| **Neutrophils (10^3^/uL)** | 8.90 | 15.24 | 4.53 | 2.21 | 3.03 |
| *SD* | *3.14* | *4.78* | *1.49* | *0.80* | *1.82* |
| **Lymphocytes (10^3^/uL)** | 5.09 | 5.40 | 5.42 | 6.11 | 7.52 |
| *SD* | *1.80* | *1.48* | *1.06* | *1.58* | *2.13* |
| **RBC (10^6^/uL)** | 4.48 | 5.53 | 5.10 | 4.17 | 4.73 |
| *SD* | *0.56* | *0.88* | *0.78* | *0.62* | *0.53* |
| **RET (%)** | 5.46 | 5.89 | 1.36 | 1.58 | 1.48 |
| *SD* | *1.32* | *1.35* | *0.60* | *0.77* | *0.57* |
| **HGB (g/dL)** | 14.66 | 17.94 | 16.14 | 12.44 | 11.10 |
| *SD* | *1.47* | *2.27* | *1.92* | *1.69* | *0.97* |
| **PLT (10^3^/uL)** | 302.37 | 244.23 | 297.19 | 262.33 | 369.22 |
| *SD* | *62.65* | *65.10* | *86.67* | *80.99* | *130.03* |

## **TableS6.** Summary of Biosensor data in neonates, infants, older children and adults. G6PD activity is expressed in U/gHb.

| **Reference** | **N** | **Age** | **Blood type** | **Median**  **G6PD by Biosensor** | **Deficient (≤30%)** | **Intermediate (31-70%)** | **Normal (>70%)** |
| --- | --- | --- | --- | --- | --- | --- | --- |
| Bancone et al.,2022 | 125 | Birth | Cord blood | 14.4* | ≤4.8^$^ | 4.9-9.9 | ≥10.0^$^ |
| Manowong et al.,2022 | 76 | 1-7 days | Whole blood | 12.7* | ≤3.6 |  |  |
| This study | 26^&^ | Birth | Cord blood | 12.10 | ≤3.6 | 3.7-8.5 | ≥8.6 |
| This study | 27^&^ | < 24H | Capillary | 12.30 | ≤3.7 | 3.8-8.6 | ≥8.7 |
| This study | 27^&^ | 7 days | Capillary | 11.60 | ≤3.5 | 3.6-8.1 | ≥8.1 |
| This study | 27^&^ | 28 days | Capillary | 10.30 | ≤3.1 | 3.2-7.2 | ≥7.3 |
| This study | 26^&^ | 4 months | Capillary | 9.35 | ≤2.8 | 2.9-6.5 | ≥6.6 |
| Manufacturer’s thresholds | Hundreds | >2 years | Venous and capillary |  | ≤4.0 | 4.1-6.0 | ≥6.1 |

* Male Median. ^$^Thresholds calculated by ROC curve analysis. ^&^ Includes only males and females with wild type genotype

# Figures

## **Figure S1.** Age at sampling of the first capillary blood sample


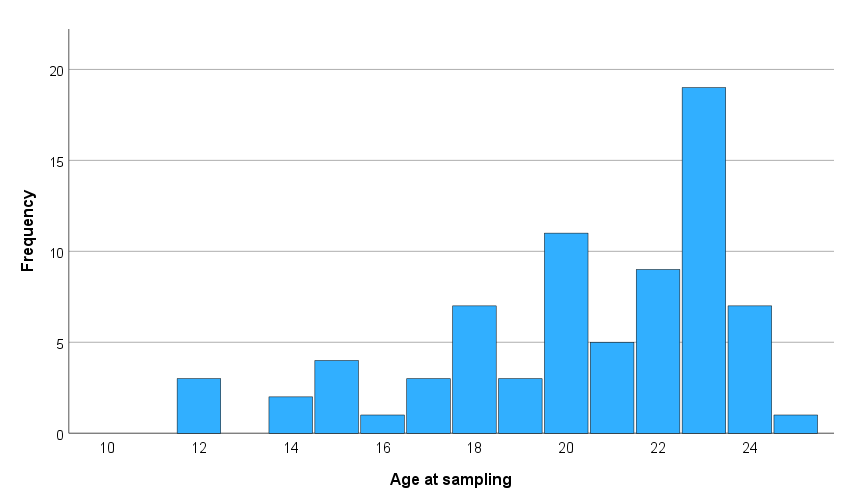


## **Figure S2.** Individual participants’ G6PD activity over time


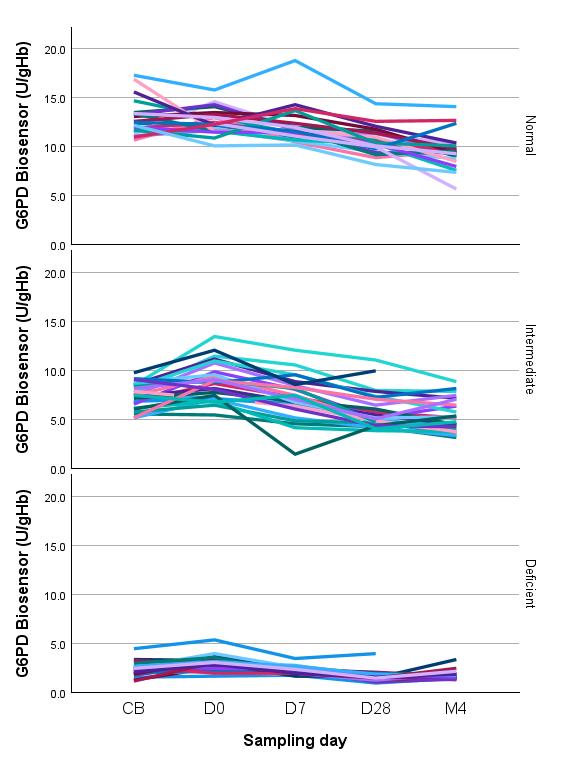


## **Figure S3.** Scatter plot of activity by Biosensor in cord blood against capillary sample (<24h of life)


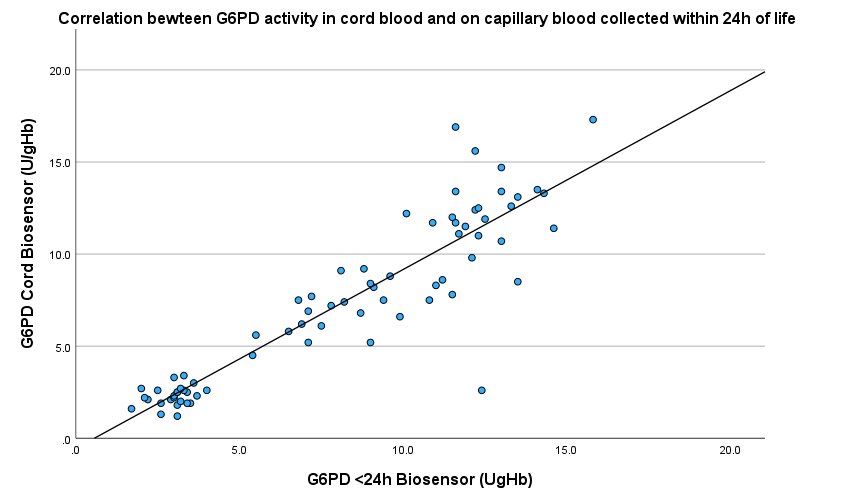


## **Figure S4.** Scatter plot of activity by Biosensor in cord blood against capillary sample (day 7)

**
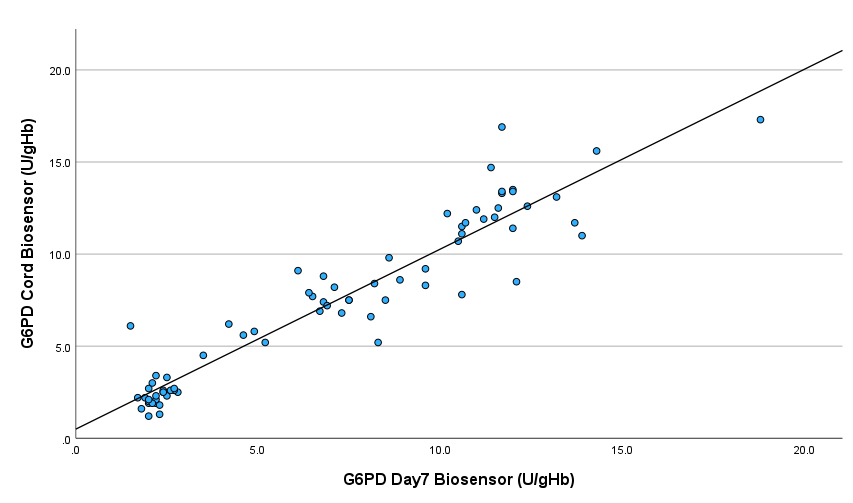
**

## **Figure S5**. Change over time by G6PD Status in: A. Haemoglobin concentration, B. White blood cells, C. Red blood cells, and D. Reticulocytes.


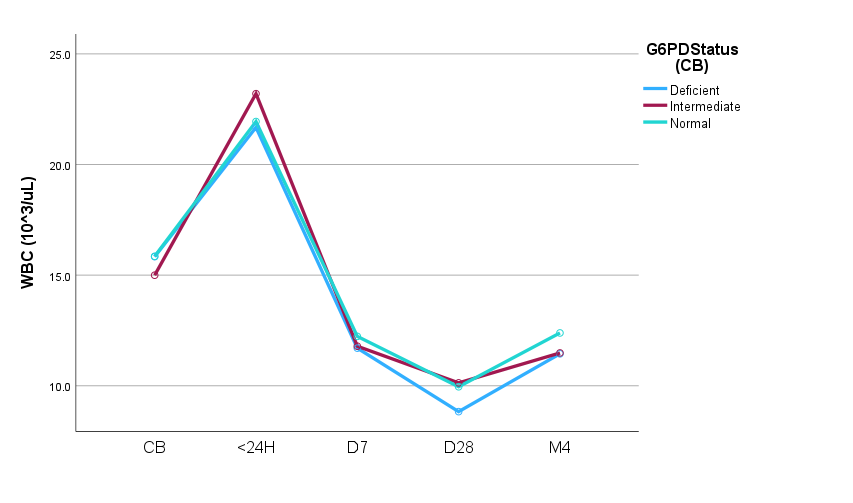

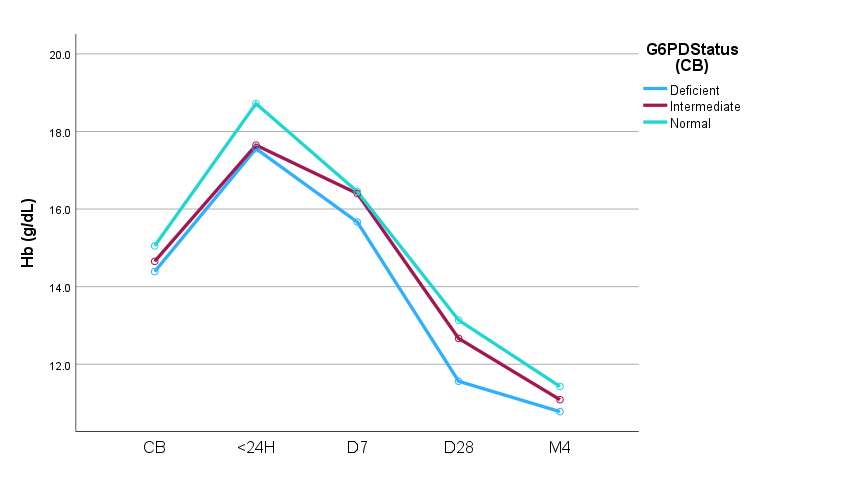

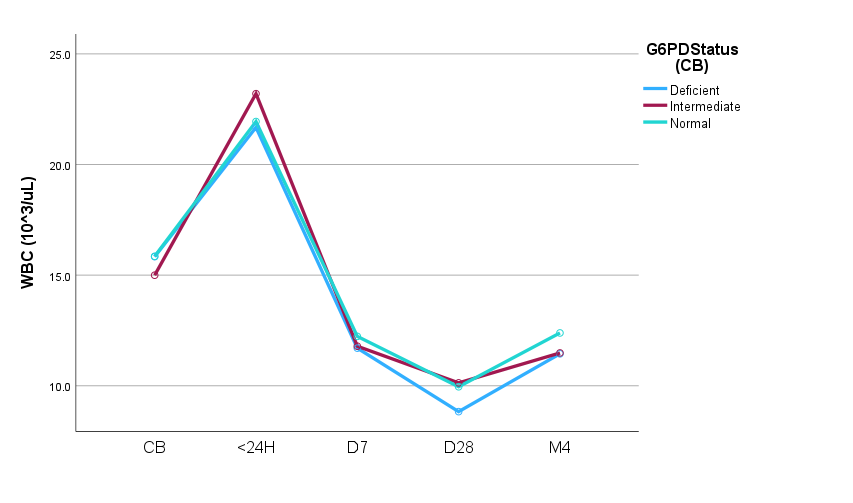
A. B.

C. D.


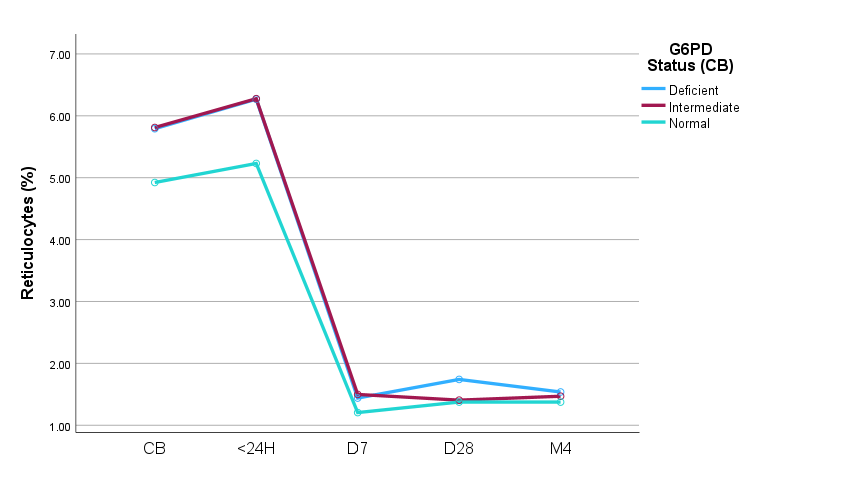

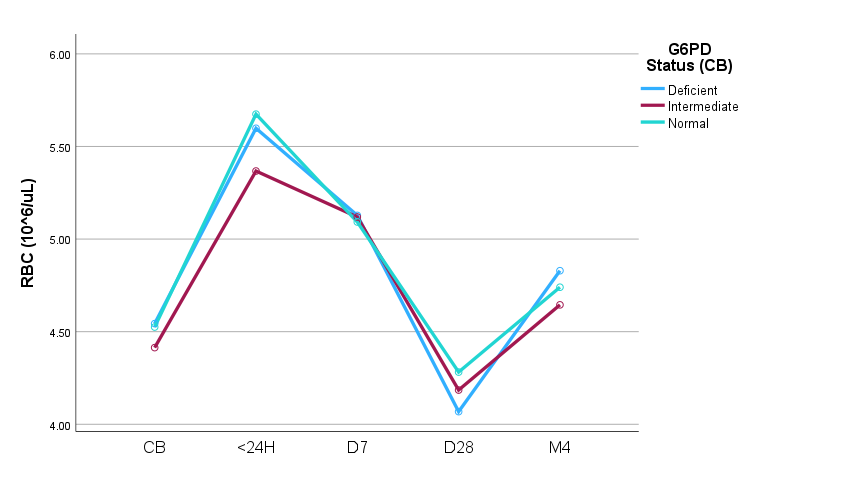


**Figure S6**. Bland-Altman plot of G6PD results in **A**. cord blood, **B**. capillary blood collected within 24h of life, **C**. Day 7, **D**. Day 28 and **E**. Month 4.

1.
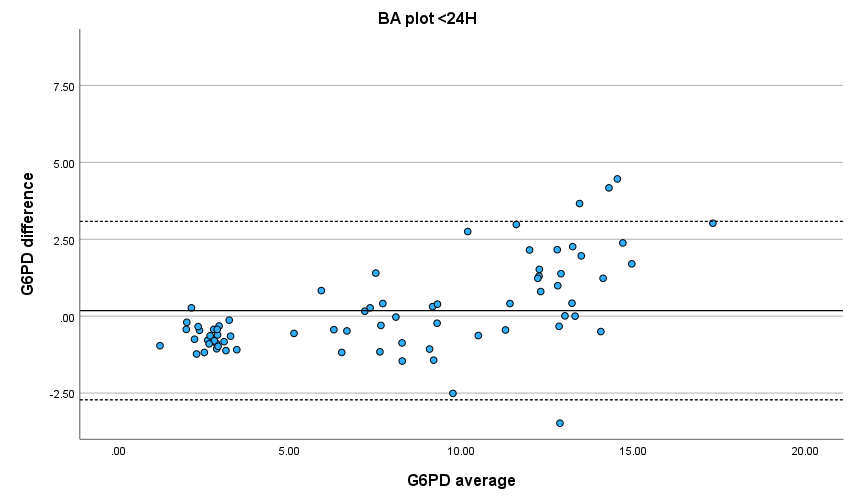
 B.


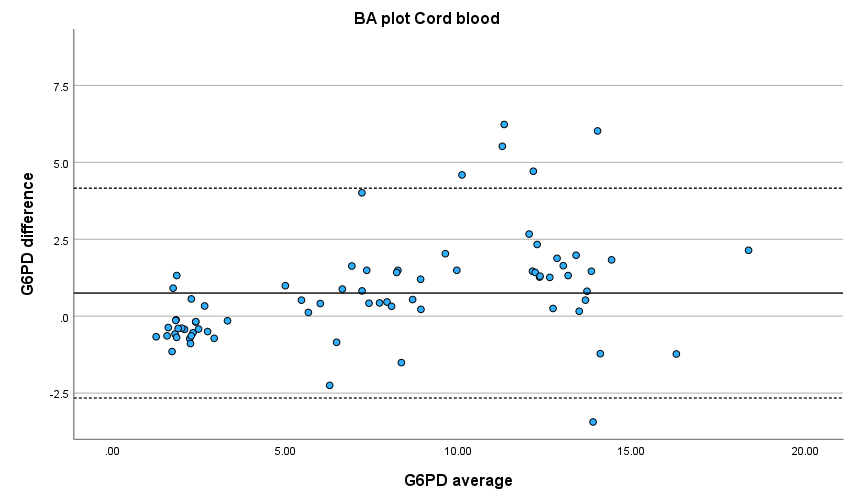


1. D.


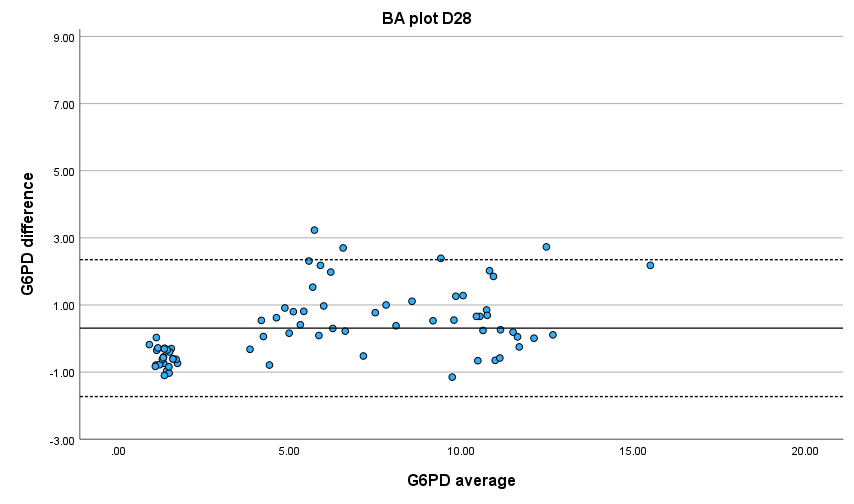

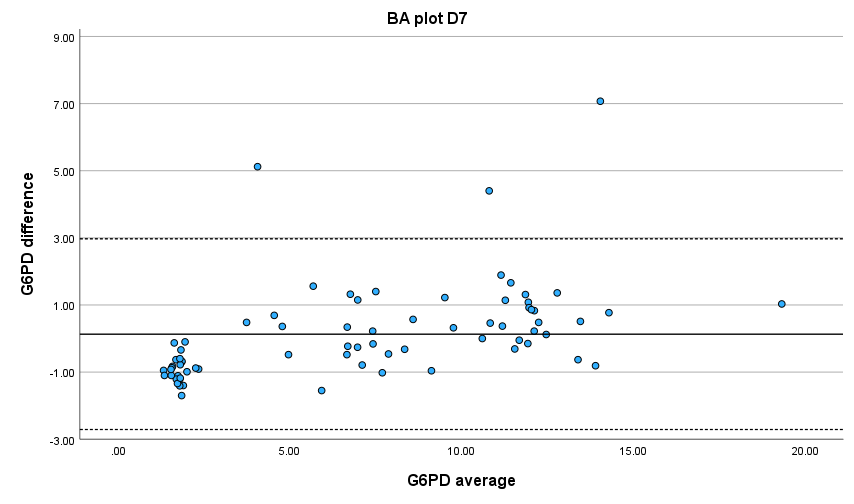


E.


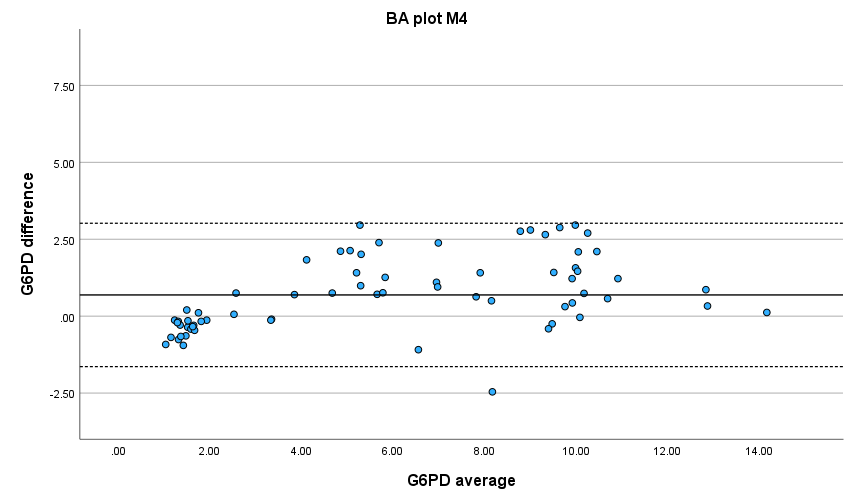


## **Figure S7.** Bland-Altman plot for haemoglobin detected by Biosensor against CBC

**
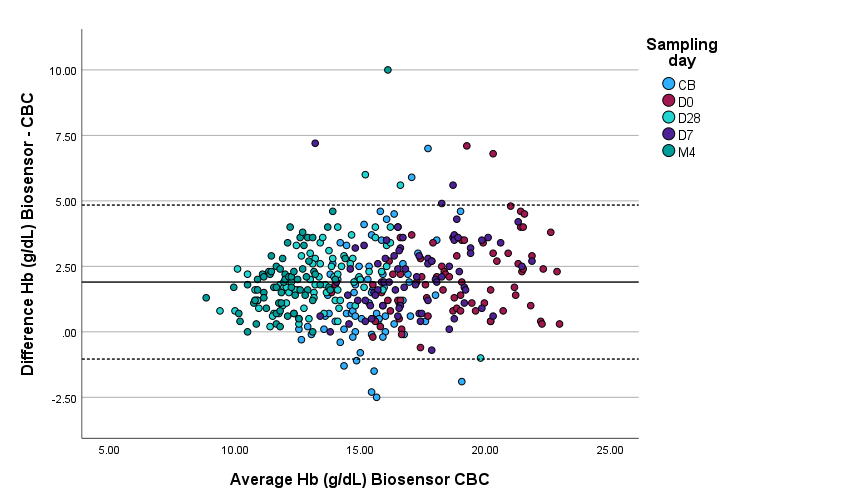
**

## **Figure S8.** Bland-Altman plot for haemoglobin detected by Biosensor against Hemocue


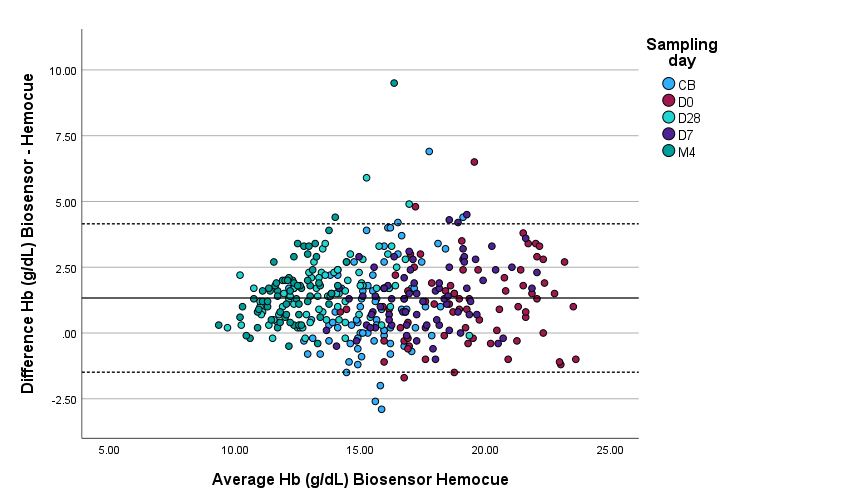

Supplement: S1 File — (DOCX) [file pone.0346837.s001.docx]
