# Supplementary material for: Use of the “STANDARD G6PDTM” quantitative point-of-care test in neonates and infants
Source: PLoS One. 2026 Jul 10;21(7):e0346837. doi: 10.1371/journal.pone.0346837 (PMC13354085; doi:10.1371/journal.pone.0346837)
Supplement: S2 File — (DOCX) [file pone.0346837.s002.docx]

**G6PD activity by Spectrophotometry: comparison between cord blood and follow-up capillary samples**

Overall results of G6PD activity according to phenotypic group using Spectrophotometry are reported in Table A1.

**Table A1.** **Median (min-max) G6PD activity by sampling day using the Spectrophotometer (IU/gHb) according to spectrophotometry-defined G6PD phenotype established at birth.**

|  | | Cord blood | <H24 | D7 | D28 | M4 |
| --- | --- | --- | --- | --- | --- | --- |
| Deficient | N | 25 | 25 | 25 | 25 | 24 |
|  | **Median** | **1.9** | **2.4 (126%)** | **1.2 (63%)** | **1.0 (53%)** | **1.4 (74%)** |
|  | Minimum | 0.9 | 0.7 | 0.8 | 0.7 | 0.6 |
|  | Maximum | 3.3 | 3.2 | 1.9 | 1.4 | 3.3 |
| Intermediate | N | 20 | 20 | 20 | 20 | 17 |
|  | **Median** | **7.7** | **7.7 (100%)** | **6.8 (88%)** | **6.1 (79%)** | **6.1 (79%)** |
|  | Minimum | 5.2 | 4.8 | 4.0 | 3.7 | 3.3 |
|  | Maximum | 9.2 | 10.2 | 8.9 | 7.9 | 8.1 |
| Normal | N | 29 | 30 | 30 | 30 | 29 |
|  | **Median** | **13.6*** | **13.2 (97%)** | **12.4 (91%)** | **10.8 (79%)** | **10.5 (77%)** |
|  | Minimum | 9.5 | 8.5 | 6.8 | 5.5 | 6.0 |
|  | Maximum | 19.4 | 18.8 | 19.8 | 16.6 | 14.2 |

* Male median G6PD activity assessed before in cord blood was 13.3

Pearson’s correlation coefficient between G6PD in cord blood and in capillary sample collected within 24h from birth was 0.980 (p<0.01) when analysed by spectrophotometer. Overall G6PD activity in paired samples was significantly lower in capillary blood from day 7 compared with cord blood when analysed by spectrophotometry (P<0.01). G6PD activity in paired samples was significantly higher at 24 hours compared with cord blood in capillary blood of deficient participants but not intermediate and normal participants. When using spectrophotometry, two participants (1 intermediate and 1 normal) would have been classified differently using capillary blood within 24 hours as compared to cord blood (highlighted in red in Table A2, left pane) and 2 G6PD normal who would have been categorized differently using capillary blood at day 7 (Table A2, right pane).

**Table A2. Comparison of G6PD status by spectrophotometry (using spectrophotometric CB thresholds)**

| G6PD status | G6PD Status (<24H) | | | G6PD Status (D7) | | |
| --- | --- | --- | --- | --- | --- | --- |
| Cord blood | Deficient | Intermediate | Normal | Deficient | Intermediate | Normal |
| Deficient (25) | 25 | 0 | 0 | 25 | 0 | 0 |
| Intermediate (20) | 0 | 19 | **1** | **1** | 19 | 0 |
| Normal (29) | 0 | **1** | 28 | 0 | **2** | 27 |

Numbers in bold indicate misclassification of phenotype in the participant.
