# Supplementary material for: Use of the “STANDARD G6PDTM” quantitative point-of-care test in neonates and infants
Source: PLoS One. 2026 Jul 10;21(7):e0346837. doi: 10.1371/journal.pone.0346837 (PMC13354085; doi:10.1371/journal.pone.0346837)
Supplement: Attachment — Submitted filename: Review PONE_D_26-14074.docx [file pone.0346837.s003.docx]

## Review of the manuscript: ‘Evaluation of diagnostic performance of the “STANDARD G6PD™” quantitative point-of-care test in neonates and infants.’ (PONE-D-26-14074)

### Major Comments

- The abstract and discussion sections mentioned performance, sensitivity, and specificity, but these were not described in the methods and results sections. Please describe how the performance was calculated, the definition of True Positives, True Negatives, etc., and include their results.
- Many of the comparisons in the result section were stated without their p-values, which omitted valuable information
- Some of the comparisons in the result section were unclear about their comparator, making it difficult to understand what was being compared and what was the definition “standard” or “correct” classification. It would be best if what was considered as the “correct” classification was described prior, in the methods section.
- The results from spectrophotometry measurements were discussed as often as the results from Biosensor, but the urgency and relation to the study aim was quite unclear.
- For example under the subheading “G6PD activity by Biosensor: comparison between cord blood and follow-up capillary samples”, many of the findings described were based on spectrophotometry measurements. I suggest a separate subheading to describe spectro results to avoid confusion

### Comments

- General comments
  - Kindly observe the use of correct English grammar throughout the text
  - Please spell out abbreviations when they are first used, followed by the abbreviation in brackets. For example, “wild type (WT)”.
- Abstract
  - Lines 49-51 “Performance of the point-of-care test as compared to the gold standard spectrophotometry remained excellent at all sampling time-points.”: I find this statement to be misleading because the performance measures of the Biosensor were not reported in the text.
  - Lines 51-52 “G6PD activity assessed longitudinally in the same participants decreased over time,”: Only the decrease of median G6PD activity was reported (Table 1 and Table 2); it would be great if the individual decrease in activities was also presented to support this statement. Please also specify that the decrease happens after the D7 measurement, following an increase in H24.
- Methods
  - Lines 113-114 “Neonates are classified based on thresholds established before”: Please cite the appropriate reference.
  - Lines 114-115 “deficient (≤4.8 IU/gHb), intermediate (4.9-9.9IU/gHb, females only) and normal (≥10.0IU/gHb).”: The classification can be clarified. For example, what will be the status of a male baby with 6 U/g Hb activity?
  - Line 169: What's the rationale behind presenting min-max instead of IQR? Also please write minimum and maximum appropriately without abbreviating.
  - Lines 169-171 “Correlation between activity in cord blood and at follow-up was assessed using Pearson’s coefficient of correlation.”: I would suggest doing a Bland-Altman analysis as well.
  - Line 171: Kindly define what was referred to as “paired samples” prior to this sentence. Did you mean cord blood vs capillary follow-up measurements from the same individual?
- Results
  - Lines 205-208: Is there a particular reason the age of the first capillary sample was presented with mean/SD instead of in a similar format with the age of the capillary samples? From Figure S1, the average age of the first capillary sample doesn't seem to be normally distributed.
  - Line 222: Spell out CBC when first using any abbreviations
  - Line 226 “a new mutation 50G>A”: Please clarify what this mutation position corresponds to (DNA? cDNA?) and which reference sequence was used to establish the position.
  - Table 3: Please spell out abbreviations when first used. If there are any abbreviations in Tables or Figures, please spell them in the Table's footnotes or Figure's caption
  - Line 235 “(p<0.01)”: Generally, p-values are written with 3 decimal points. If the p-value was between 0.01 and 0.001, please write the exact value.
  - Line 236 “G6PD activity in paired samples was significantly higher at 24 hours …”:
    - Were you referring to median G6PD activity?
    - I’m still unsure about the definition of ‘paired samples’. Were you referring to the capillary samples at 24 hours, and not actually talking about a pair of samples? Please clarify
    - If statistical tests comparing the capillary blood with the cord blood per G6PD status category were performed, please include the p-values in Table 1
  - Lines 238-239 “Activity was not different in G6PD normal participants analysed by either method”: Kindly add the p-values from the Wilcoxon Signed-Rank Test or paired T-test to support this sentence.
  - Lines 246-248 “When comparing status classification, all G6PD deficient participants but one (a deficient heterozygote) would have been correctly identified as deficient by 24-hours and day-7 capillary blood using cord blood thresholds by Biosensor (Figure 2A) and spectrophotometry.”: Please clarify what was referred to as the correct/reference identification here, was it cord blood spectrophotometry? Cord blood Biosensor?
  - Figure 2A: Could you please clarify what the asterisk signs in the graph mean? And how are they different from the outlier dots
  - Figure 2B: Please add the dotted lines for the thresholds to Fig 2 B
  - Lines 250-252 “At 24 hours, there were 6 participants with intermediate activity who would be classified differently if analysis was carried out in capillary blood rather than cord blood …”: But the cord blood was taken during birth and not at 24 hours? I suggest rewording the sentence. Were you referring to the cap blood threshold vs cord blood threshold, rather than talking about the actual samples?
  - Paragraph starting at line 246: So in this paragraph, the cord blood measurements by Biosensor and Spectro were treated as the “correct” identification? Is there any biological rationale for this? If not, I would change the term “correctly identified” in this paragraph and stress that this was just a comparison of status identification
  - Table 4 and Table 5: Please repeat in the table's footnote what the red highlights mean.
  - Line 282: Spell out abbreviation when first use (ICC)
  - Line 303 “In particular, significant increase in WBC, RBC count and reticulocyte percentage at <24h of life”: Please state the respective p-value of each increase
  - Line 304 “in all G6PD phenotypic groups”: Table S5 need to be stratified by phenotypic groups to support the findings stated in this sentence
- Discussion
  - Lines 337-338: Please add the citation using a proper format
  - Line 341 “A Second study”: Add citation
  - Line 344 “Table S6 shows results …”: Table S6 showed thresholds, not results?
  - Lines 348-351: These findings were already described in the Results section
  - Lines 353-355: There was no sensitivity or specificity among the findings described in the results section. Either describe these results or refrain from discussing them.
  - Lines 358-360 “blood indices are generally similar but tend to be higher in neonatal blood (Greer, Safarulla et al. 2019), particularly for WBC (Scheffer-Mendoza, Espinosa-Padilla et al. 2020)”: How were the results in this study compared to the results in these articles? Do they agree with each other? Are they contradictory?
  - Lines 363-365 “increased number of WBC and young reticulocytes would have impacted on total detected activity, especially in G6PD deficient and intermediate samples.”: Please add an appropriate citation
