# Supplementary material for: Use of the “STANDARD G6PDTM” quantitative point-of-care test in neonates and infants
Source: PLoS One. 2026 Jul 10;21(7):e0346837. doi: 10.1371/journal.pone.0346837 (PMC13354085; doi:10.1371/journal.pone.0346837)
Supplement: Attachment — Submitted filename: Responses_to_reviewers_final.docx [file pone.0346837.s006.docx]

Dear Editor and Reviewers,

We have thoroughly revised the manuscript and following your suggestions we have streamlined the text and made it clearer.

Major changes we made based on reviewers’ comments are:

1. Modified the Title to be more in line with the study aims
2. Added a new figure (S2) with individual G6PD activity changes over time in all the participants
3. Moved to supplementary file a large part of results about gold standard spectrophotometry
4. Modified Table 4 (now Table 3)

Below the point-to-point answers to the questions and comments raised by the reviewers.

**Reviewer 1**

Major Comments
**• The abstract and discussion sections mentioned performance, sensitivity, and specificity, but these were not described in the methods and results sections. Please describe how the performance was calculated, the definition of True Positives, True Negatives, etc., and include their results.**

We appreciate the wording of title and abstract might have been confusing but the device has been already fully evaluated before. The title included “performance” meant in the broader sense to include all study aims explained in the abstract and introduction *(“The primary aim of the study was to assess equivalency of cord blood and neonatal capillary blood for diagnosis of G6PD deficiency using the quantitative point-of-care “STANDARD G6PD^TM^” test (SD Biosensor, Korea). Additional secondary aims were to compare the “STANDARD G6PD^TM^” with gold standard spectrophotometry and to analyse changes in G6PD activity in the first 4 months of life.”*)

The abstract does not mention sensitivity and specificity explicitly, but following the reviewers’ comments, we have modified the title and the abstract.

• **Many of the comparisons in the result section were stated without their p-values, which omitted valuable information**

These have now been added

• **Some of the comparisons in the result section were unclear about their comparator, making it difficult to understand what was being compared and what was the definition “standard” or “correct” classification. It would be best if what was considered as the “correct” classification was described prior, in the methods section.**

The reference classification used in the manuscript was reported in both the methods (line 110-112) and again in the results (192-194).

**• The results from spectrophotometry measurements were discussed as often as the results from Biosensor, but the urgency and relation to the study aim was quite unclear.**

The main objective of the study was not to re-evaluate the performance of the device against the spectrophotometry and the study was not powered for this. But since the spectrophotometry was carried out in all samples, we decided to show the results (using Bland-Altman plots) to confirm that performance of the device did not deviate from what already observed in cord blood and children/adult blood. In order to make the manuscript more concise and focused, and following the reviewers’ suggestions, we have decided to move results of spectrophotometric analyses to supplementary material to avoid confusion (see also later).

**• For example under the subheading “G6PD activity by Biosensor: comparison between cord blood and follow-up capillary samples”, many of the findings described were based on spectrophotometry measurements. I suggest a separate subheading to describe spectro results to avoid confusion**

Thanks for the suggestion. We have revised the result section and moved the results of the spectrophotometer in the supplementary section to avoid confusion

**Comments
• General comments
o Kindly observe the use of correct English grammar throughout the text**

We have revised the manuscript for English grammar
o **Please spell out abbreviations when they are first used, followed by the abbreviation in brackets. For example, “wild type (WT)”.**

This was done
**• Abstract
o Lines 49-51 “Performance of the point-of-care test as compared to the gold standard spectrophotometry remained excellent at all sampling time-points.”: I find this statement to be misleading because the performance measures of the Biosensor were not reported in the text.**

We have re-phrased as “Agreement of G6PD activity of the point-of-care test as compared to the gold standard spectrophotometry remained excellent at all sampling time-points.” These data are reported in Table 6 and in Bland Altman graphs in Figure S9 to S13

**o Lines 51-52 “G6PD activity assessed longitudinally in the same participants decreased over time,”: Only the decrease of median G6PD activity was reported (Table 1 and Table 2); it would be great if the individual decrease in activities was also presented to support this statement. Please also specify that the decrease happens after the D7 measurement, following an increase in H24.**

As shown and explained in much details later, the activity increases at H24 but then decreases from 1 week to 4 months. Line 51-52 is a general statement in the abstract which reflects the overall (cord blood to 4 month) time course.

In terms of showing individual activities changes over time, we have now added a new Figure (Figure S2) in supplementary file 1.

**• Methods
o Lines 113-114 “Neonates are classified based on thresholds established before”: Please cite the appropriate reference.**

This was added

**o Lines 114-115 “deficient (≤4.8 IU/gHb), intermediate (4.9-9.9IU/gHb, females only) and normal (≥10.0IU/gHb).”: The classification can be clarified. For example, what will be the status of a male baby with 6 U/g Hb activity?**

This has been clarified.

**o Line 169: What's the rationale behind presenting min-max instead of IQR? Also please write minimum and maximum appropriately without abbreviating.**

We have corrected the appropriate spelling of minimum and maximum throughout the manuscript. The rationale for presenting minimum and maximum lays in the use of activity categorizations; we believe that reporting minimum and maximum values recorded at each time point can give a better indication of how spread results were and how many were outside the pre-established cut-offs.

**o Lines 169-171 “Correlation between activity in cord blood and at follow-up was assessed using Pearson’s coefficient of correlation.”: I would suggest doing a Bland-Altman analysis as well.**

We have to disagree with the reviewer on this point as Bland-Altman analysis is not appropriate for this type of analysis. We have used it instead when comparing results from different tests carried out in the same sample.

**o Line 171: Kindly define what was referred to as “paired samples” prior to this sentence. Did you mean cord blood vs capillary follow-up measurements from the same individual?**

Yes, paired samples were samples collected over time in the same individual.

**• Results
o Lines 205-208: Is there a particular reason the age of the first capillary sample was presented with mean/SD instead of in a similar format with the age of the capillary samples? From Figure S1, the average age of the first capillary sample doesn't seem to be normally distributed.**

As highlighted by the reviewer, mean/SD was not the correct way to show the age of first capillary sample and it has now been corrected.

The age is not normally distributed because the indication was to collect the sample within the first 24h of life, as close as possible to the 24h mark; considerations about reducing the number of needle sticks in neonates who needed blood for clinical reasons and avoiding nightly collections were also taken into account, therefore some of the blood samples were collected much earlier than 24 hours.

**o Line 222: Spell out CBC when first using any abbreviations**

This was done

**o Line 226 “a new mutation 50G>A”: Please clarify what this mutation position corresponds to (DNA? cDNA?) and which reference sequence was used to establish the position.**

This is the position of the coding DNA; reference sequence is ENSG00000160211 (transcript G6PD-006). This was added to the manuscript

**o Table 3: Please spell out abbreviations when first used. If there are any abbreviations in Tables or Figures, please spell them in the Table's footnotes or Figure's caption**

This was done

**o Line 235 “(p<0.01)”: Generally, p-values are written with 3 decimal points. If the p-value was between 0.01 and 0.001, please write the exact value.**

This was done

**o Line 236 “G6PD activity in paired samples was significantly higher at 24 hours …”:
§ Were you referring to median G6PD activity?
§ I’m still unsure about the definition of ‘paired samples’. Were you referring to the capillary samples at 24 hours, and not actually talking about a pair of samples? Please clarify
§ If statistical tests comparing the capillary blood with the cord blood per G6PD status category were performed, please include the p-values in Table 1**

This was included in the text at Line 232-238 in the revised version

**o Lines 238-239 “Activity was not different in G6PD normal participants analysed by either method”: Kindly add the p-values from the Wilcoxon Signed-Rank Test or paired T-test to support this sentence**.

P-values from the Wilcoxon Signed-Rank Test were added for all comparisons

**o Lines 246-248 “When comparing status classification, all G6PD deficient participants but one (a deficient heterozygote) would have been correctly identified as deficient by 24-hours and day-7 capillary blood using cord blood thresholds by Biosensor (Figure 2A) and spectrophotometry.”: Please clarify what was referred to as the correct/reference identification here, was it cord blood spectrophotometry? Cord blood Biosensor?**

This referred to only Biosensor, the words “and spectrophotometry” were a mistake (since the data for spectrophotometry were reported later). We have corrected the text and moved the spectrophotometry section to the supplementary file 2.

**o Figure 2A: Could you please clarify what the asterisk signs in the graph mean? And how are they different from the outlier dots**

In box-plots, SPSS used asterisk to indicate extreme outliers (>3 time the height of the box) as compared to circles which indicate outliers (values that do no fall in the inner fences). This has been added to the caption.

**o Figure 2B: Please add the dotted lines for the thresholds to Fig 2 B**

There are no dotted lines in this Figure because G6PD genotypes in females are not expected to be associated with any specific activity range

**o Lines 250-252 “At 24 hours, there were 6 participants with intermediate activity who would be classified differently if analysis was carried out in capillary blood rather than cord blood …”: But the cord blood was taken during birth and not at 24 hours? I suggest rewording the sentence. Were you referring to the cap blood threshold vs cord blood threshold, rather than talking about the actual samples?**

This indicates that using the cord-blood derived thresholds for classification of activity in capillary blood collected at 24h would result in misclassification of 6 participants. This has been re-phrased for clarity.

The way this analysis was carried out is explained in the methods.

**o Paragraph starting at line 246: So in this paragraph, the cord blood measurements by Biosensor and Spectro were treated as the “correct” identification? Is there any biological rationale for this? If not, I would change the term “correctly identified” in this paragraph and stress that this was just a comparison of status identification**

The primary aim of the study was to see whether thresholds already validated in cord blood could be used in capillary blood collected during the first week of life. Therefore, the classification of participants based on the cord blood is considered the correct identification.

Practically, this has large impact on the way G6PD activity can be tested in neonates and high risk of jaundice be identified.

**o Table 4 and Table 5: Please repeat in the table's footnote what the red highlights mean.**

This was done

**o Line 282: Spell out abbreviation when first use (ICC)**

This was done

**o Line 303 “In particular, significant increase in WBC, RBC count and reticulocyte percentage at <24h of life”: Please state the respective p-value of each increase**

Added
**o Line 304 “in all G6PD phenotypic groups”: Table S5 need to be stratified by phenotypic groups to support the findings stated in this sentence**

There is no difference by phenotypic groups as clearly visible in Figure S5 A to D; the comparison is between cord blood and 24h sample, not among different phenotypic group. All comparisons are statistically significant at the p<0.001 level aside from reticulocytes in G6PD normal where the p is 0.003

**• Discussion
o Lines 337-338: Please add the citation using a proper format**

This has been corrected

**o Line 341 “A Second study”: Add citation**

The reference was added
**o Line 344 “Table S6 shows results …”: Table S6 showed thresholds, not results?**

We have modified this sentence
**o Lines 348-351: These findings were already described in the Results section**

Yes this findings were already described in results and they were redundant. We have delete them here.
**o Lines 353-355: There was no sensitivity or specificity among the findings described in the results section. Either describe these results or refrain from discussing them.**

We think a consideration about the lack of sensitivity or specificity can be made based on available data even without a formalized sensitivity and specificity analysis.

**o Lines 358-360 “blood indices are generally similar but tend to be higher in neonatal blood (Greer, Safarulla et al. 2019), particularly for WBC (Scheffer-Mendoza, Espinosa-Padilla et al. 2020)”: How were the results in this study compared to the results in these articles? Do they agree with each other? Are they contradictory?**

They all go on the same direction. We have re-phrased for clarity.

**o Lines 363-365 “increased number of WBC and young reticulocytes would have impacted on total detected activity, especially in G6PD deficient and intermediate samples.”: Please add an appropriate citation**

Two citations were added.

**Reviewer 2**

**The authors present a relatively quick and effective diagnostic PoC test for neonatal G6PD quantitation. The work is succinctly presented and the manuscript itself is well-written. Adding a few comments can help improve the depth of the manuscript:
1. The authors present a good discussion on the correlation between sex and diagnostic threshold. In tables 3-5, presenting the data in correlation with the sex of the neonate can be helpful in drawing more conclusions.**

This is a good suggestion and usually we would show data separately for males and females. In this case we are dealing mostly with G6PD phenotypes and the associated risk of jaundice which is directly linked to the phenotype (regardless of the sex). For this reason, we feel that following a phenotypic categorization is more appropriate.

**2. How sensitive is the accuracy of G6PD-deficiency classification if the diagnostic thresholds were to be adjusted for (i) different time points, and (ii) cord blood v/s capillary blood? Please comment.**

While such an analysis might be intriguing, the study was not designed to assess sensitivity of different diagnostic thresholds at different time points so, unfortunately we cannot answer this question.

**3. The authors should talk more about the reliability of this test in deficient patients with novel and/or rare mutations.**

In this study we only found a novel mutation in a heterozygote participant so we do not know what level of residual activity is associated to the mutation in hemizygote state. Since the test assesses G6PD activity and not genotype, we would expect the reliability be the same across genotypes.

**Reviewer 3**

**Consider whether the information presented in this paper can be consolidated without compromising your conclusions. The results section, in particular, is quite lengthy, and trimming the wordcount might improve readability.**

We agree with the reviewer and have decided to move the comparisons of activity by spectrophotometry to supplementary file 2.

**- Consider whether it may be appropriate to separate your discussion of analysis and results in males vs. females by sex, as the current discussion is somewhat confusing and does not seem to be clearly reflected in the tables.**

This is a good suggestion but since we are dealing mostly with phenotypes, and risk of jaundice is directly linked to phenotype, we feel that following a phenotypic categorization is more appropriate.

- **Please include units in the tables.**

Where missing, units have been included in the titles or Tables in both main Table and supplementary files

**- A discussion about costs of acquiring and using the STANDARD G6PD Biosensor test would be useful when discussing more widespread implementation of this test.**

A discussion about the cost of using the STANDARD G6PD Biosensor and how it could help decrease clinical sequelae associated with neonatal jaundice would be very interesting but unfortunately it goes beyond the scope of the study.

**- I would suggest moving the discussion paragraph spanning lines 357-365 earlier in the discussion, as it provides useful context for the rest of the information presented**.

We agree with the reviewer and have moved this paragraph earlier in the discussion

**Reviewer 4**

**The primary aim is not consistent to the title. Secondary aim is more suited with the title, therefore should be switched with the primary aim, to better suit the title**

We agree with reviewer that the primary aim is not mentioned in the title but we had chosen a broader title that could include all aims. Considering also comments from other reviewers, we have revised the title to make it even more general.

**2. Please add stronger implication on why "STANDARD-G6PD" tool is chosen to be compared to the gold standard**

This is the only commercially available quantitative POC tool and has already been evaluated for use in cord blood before in the same setting. We have added a sentence to the introduction.

**3. Please add chosen method of statistical analysis in the methods of abstract**

The abstract is already quite long, we would prefer not to include statistical methods in the abstract.

**4. Related to point (1), conclusion does answer the objectives. However, it mainly answers secondary aim, not the primary one**

In the discussion we have discussed all the aims of the study

**5. References has not been written in superscript mode, at the end of each word. The reference list at the end should be listen in numbers**

Thank you for spotting this, we are now using the correct Plos One reference style.

**Reviewer 5**

**1.The data analysis is problematic: the sample sizes are inconsistent across the tables.for example, the numbers of samples reported in Tables 3, 4, and 5 do not match.**

Thanks for spotting the error in Table 3 which has now been corrected.

Table 4 and 5 reported data only on participants with full follow-up as explained in the methods so the numbers did not represent the full sample size. However, since the only missing follow-up visit was at months 4 and not on the data points shown, we have revised the Tables and the methods. Also, Table 4 is now Table 3 and Table 5 has been moved to supplementary file 2.

**2. The clinical issues are unclear, and there are design flaws. This situation more closely resembles the design of a diagnostic trial. Why is it based on the G6PD correlation between peripheral blood and cord blood to prove that the new detection method is more effective?**

As explained in the introduction, the Biosensor has already been validated for use in cord blood and for use in capillary blood of older children and adults; we did not want to test whether the Biosensor is more effective. The question we tried to answer was about the use of neonatal capillary blood for detection of abnormal G6PD using the same cut-offs already established in cord blood. This has large practical implications for use of the device in clinical settings because it can support use of the tool not only on cord blood but also in the first few days after birth.

Several parts of the manuscript have been re-worded and we think the manuscript is now clearer.

**3.That table also has issues. The standard is a three-column table.**

We are not sure which Table the reviewer is referring to

**4. The study design lacks a work efficiency curve analysis comparing the experimental group and the gold standard. No cost comparison analysis is provided in the article, which prevents the conclusions from being effectively validated.**

We are not familiar with the “work efficiency curve analysis” mentioned by the reviewer but we have used statistics such ICC and Bland-Altman which are usually used for this type of studies.

A cost analysis, including the cost of using the STANDARD G6PD Biosensor and the saved costs associated to decrease clinical sequelae from neonatal jaundice, would be very interesting but it was unfortunately beyond the scope of this work.
