# Supplementary material for: Use of the “STANDARD G6PDTM” quantitative point-of-care test in neonates and infants
Source: PLoS One. 2026 Jul 10;21(7):e0346837. doi: 10.1371/journal.pone.0346837 (PMC13354085; doi:10.1371/journal.pone.0346837)
Supplement: Peer review history

# Peer review: Decision Letter 0

**DOI:** 10.1371/journal.pone.0346837.r001

**Authors:** Samuel Tchum

12 May 2026

PONE-D-26-14074

Evaluation of diagnostic performance of the “STANDARD G6PDTM” quantitative point-of-care test in neonates and infants

PLOS One

Dear Dr. Bancone,

Thank you for submitting your manuscript to PLOS ONE. After careful consideration, we feel that it has merit but does not fully meet PLOS ONE’s publication criteria as it currently stands. Therefore, we invite you to submit a revised version of the manuscript that addresses the points raised during the review process.

**Reviewer 1**

Major Comments

• The abstract and discussion sections mentioned performance, sensitivity, and specificity, but these were not described in the methods and results sections. Please describe how the performance was calculated, the definition of True Positives, True Negatives, etc., and include their results.

• Many of the comparisons in the result section were stated without their p-values, which omitted valuable information

• Some of the comparisons in the result section were unclear about their comparator, making it difficult to understand what was being compared and what was the definition “standard” or “correct” classification. It would be best if what was considered as the “correct” classification was described prior, in the methods section.

• The results from spectrophotometry measurements were discussed as often as the results from Biosensor, but the urgency and relation to the study aim was quite unclear.

• For example under the subheading “G6PD activity by Biosensor: comparison between cord blood and follow-up capillary samples”, many of the findings described were based on spectrophotometry measurements. I suggest a separate subheading to describe spectro results to avoid confusion

Comments

• General comments

o Kindly observe the use of correct English grammar throughout the text

o Please spell out abbreviations when they are first used, followed by the abbreviation in brackets. For example, “wild type (WT)”.

• Abstract

o Lines 49-51 “Performance of the point-of-care test as compared to the gold standard spectrophotometry remained excellent at all sampling time-points.”: I find this statement to be misleading because the performance measures of the Biosensor were not reported in the text.

o Lines 51-52 “G6PD activity assessed longitudinally in the same participants decreased over time,”: Only the decrease of median G6PD activity was reported (Table 1 and Table 2); it would be great if the individual decrease in activities was also presented to support this statement. Please also specify that the decrease happens after the D7 measurement, following an increase in H24.

• Methods

o Lines 113-114 “Neonates are classified based on thresholds established before”: Please cite the appropriate reference.

o Lines 114-115 “deficient (≤4.8 IU/gHb), intermediate (4.9-9.9IU/gHb, females only) and normal (≥10.0IU/gHb).”: The classification can be clarified. For example, what will be the status of a male baby with 6 U/g Hb activity?

o Line 169: What's the rationale behind presenting min-max instead of IQR? Also please write minimum and maximum appropriately without abbreviating.

o Lines 169-171 “Correlation between activity in cord blood and at follow-up was assessed using Pearson’s coefficient of correlation.”: I would suggest doing a Bland-Altman analysis as well.

o Line 171: Kindly define what was referred to as “paired samples” prior to this sentence. Did you mean cord blood vs capillary follow-up measurements from the same individual?

• Results

o Lines 205-208: Is there a particular reason the age of the first capillary sample was presented with mean/SD instead of in a similar format with the age of the capillary samples? From Figure S1, the average age of the first capillary sample doesn't seem to be normally distributed.

o Line 222: Spell out CBC when first using any abbreviations

o Line 226 “a new mutation 50G>A”: Please clarify what this mutation position corresponds to (DNA? cDNA?) and which reference sequence was used to establish the position.

o Table 3: Please spell out abbreviations when first used. If there are any abbreviations in Tables or Figures, please spell them in the Table's footnotes or Figure's caption

o Line 235 “(p<0.01)”: Generally, p-values are written with 3 decimal points. If the p-value was between 0.01 and 0.001, please write the exact value.

o Line 236 “G6PD activity in paired samples was significantly higher at 24 hours …”:

§ Were you referring to median G6PD activity?

§ I’m still unsure about the definition of ‘paired samples’. Were you referring to the capillary samples at 24 hours, and not actually talking about a pair of samples? Please clarify

§ If statistical tests comparing the capillary blood with the cord blood per G6PD status category were performed, please include the p-values in Table 1

o Lines 238-239 “Activity was not different in G6PD normal participants analysed by either method”: Kindly add the p-values from the Wilcoxon Signed-Rank Test or paired T-test to support this sentence.

o Lines 246-248 “When comparing status classification, all G6PD deficient participants but one (a deficient heterozygote) would have been correctly identified as deficient by 24-hours and day-7 capillary blood using cord blood thresholds by Biosensor (Figure 2A) and spectrophotometry.”: Please clarify what was referred to as the correct/reference identification here, was it cord blood spectrophotometry? Cord blood Biosensor?

o Figure 2A: Could you please clarify what the asterisk signs in the graph mean? And how are they different from the outlier dots

o Figure 2B: Please add the dotted lines for the thresholds to Fig 2 B

o Lines 250-252 “At 24 hours, there were 6 participants with intermediate activity who would be classified differently if analysis was carried out in capillary blood rather than cord blood …”: But the cord blood was taken during birth and not at 24 hours? I suggest rewording the sentence. Were you referring to the cap blood threshold vs cord blood threshold, rather than talking about the actual samples?

o Paragraph starting at line 246: So in this paragraph, the cord blood measurements by Biosensor and Spectro were treated as the “correct” identification? Is there any biological rationale for this? If not, I would change the term “correctly identified” in this paragraph and stress that this was just a comparison of status identification

o Table 4 and Table 5: Please repeat in the table's footnote what the red highlights mean.

o Line 282: Spell out abbreviation when first use (ICC)

o Line 303 “In particular, significant increase in WBC, RBC count and reticulocyte percentage at <24h of life”: Please state the respective p-value of each increase

o Line 304 “in all G6PD phenotypic groups”: Table S5 need to be stratified by phenotypic groups to support the findings stated in this sentence

• Discussion

o Lines 337-338: Please add the citation using a proper format

o Line 341 “A Second study”: Add citation

o Line 344 “Table S6 shows results …”: Table S6 showed thresholds, not results?

o Lines 348-351: These findings were already described in the Results section

o Lines 353-355: There was no sensitivity or specificity among the findings described in the results section. Either describe these results or refrain from discussing them.

o Lines 358-360 “blood indices are generally similar but tend to be higher in neonatal blood (Greer, Safarulla et al. 2019), particularly for WBC (Scheffer-Mendoza, Espinosa-Padilla et al. 2020)”: How were the results in this study compared to the results in these articles? Do they agree with each other? Are they contradictory?

o Lines 363-365 “increased number of WBC and young reticulocytes would have impacted on total detected activity, especially in G6PD deficient and intermediate samples.”: Please add an appropriate citation

**Reviewer 2**

The authors present a relatively quick and effective diagnostic PoC test for neonatal G6PD quantitation. The work is succinctly presented and the manuscript itself is well-written. Adding a few comments can help improve the depth of the manuscript:

1. The authors present a good discussion on the correlation between sex and diagnostic threshold. In tables 3-5, presenting the data in correlation with the sex of the neonate can be helpful in drawing more conclusions.

2. How sensitive is the accuracy of G6PD-deficiency classification if the diagnostic thresholds were to be adjusted for (i) different time points, and (ii) cord blood v/s capillary blood? Please comment.

3. The authors should talk more about the reliability of this test in deficient patients with novel and/or rare mutations.

**Reviewer 3**

Consider whether the information presented in this paper can be consolidated without compromising your conclusions. The results section, in particular, is quite lengthy, and trimming the wordcount might improve readability.

- Consider whether it may be appropriate to separate your discussion of analysis and results in males vs. females by sex, as the current discussion is somewhat confusing and does not seem to be clearly reflected in the tables.

- Please include units in the tables.

- A discussion about costs of acquiring and using the STANDARD G6PD Biosensor test would be useful when discussing more widespread implementation of this test.

- I would suggest moving the discussion paragraph spanning lines 357-365 earlier in the discussion, as it provides useful context for the rest of the information presented.

**Reviewer 4**

The primary aim is not consistent to the title. Secondary aim is more suited with the title, therefore should be switched with the primary aim, to better suit the title

2. Please add stronger implication on why "STANDARD-G6PD" tool is chosen to be compared to the gold standard

3. Please add chosen method of statistical analysis in the methods of abstract

4. Related to point (1), conclusion does answer the objectives. However, it mainly answers secondary aim, not the primary one

5. References has not been written in superscript mode, at the end of each word. The reference list at the end should be listen in numbers

**Reviewer 5**

1. The data analysis is problematic: the sample sizes are inconsistent across the tables.for example, the numbers of samples reported in Tables 3, 4, and 5 do not match.

2. The clinical issues are unclear, and there are design flaws. This situation more closely resembles the design of a diagnostic trial. Why is it based on the G6PD correlation between peripheral blood and cord blood to prove that the new detection method is more effective?

3.That table also has issues. The standard is a three-column table.

4. The study design lacks a work efficiency curve analysis comparing the experimental group and the gold standard. No cost comparison analysis is provided in the article, which prevents the conclusions from being effectively validated.

Please submit your revised manuscript by Jun 26 2026 11:59PM. If you will need more time than this to complete your revisions, please reply to this message or contact the journal office at plosone@plos.org. When you're ready to submit your revision, log on to https://www.editorialmanager.com/pone/ and select the 'Submissions Needing Revision' folder to locate your manuscript file.

Please include the following items when submitting your revised manuscript:

- A letter that responds to each point raised by the academic editor and reviewer(s). You should upload this letter as a separate file labeled 'Response to Reviewers'.
- A marked-up copy of your manuscript that highlights changes made to the original version. You should upload this as a separate file labeled 'Revised Manuscript with Track Changes'.
- An unmarked version of your revised paper without tracked changes. You should upload this as a separate file labeled 'Manuscript'.

If you would like to make changes to your financial disclosure, please include your updated statement in your cover letter. Guidelines for resubmitting your figure files are available below the reviewer comments at the end of this letter.

If applicable, we recommend that you deposit your laboratory protocols in protocols.io to enhance the reproducibility of your results. Protocols.io assigns your protocol its own identifier (DOI) so that it can be cited independently in the future. For instructions see: https://journals.plos.org/plosone/s/submission-guidelines#loc-laboratory-protocols. Additionally, PLOS ONE offers an option for publishing peer-reviewed Lab Protocol articles, which describe protocols hosted on protocols.io. Read more information on sharing protocols at https://plos.org/protocols?utm_medium=editorial-email&utm_source=authorletters&utm_campaign=protocols.

As the corresponding author, your ORCID iD is verified in the submission system and will appear in the published article. PLOS supports the use of ORCID, and we encourage all coauthors to register for an ORCID iD and use it as well. Please encourage your coauthors to verify their ORCID iD within the submission system before final acceptance, as unverified ORCID iDs will not appear in the published article. *Only*  the individual author can complete the verification step; PLOS staff *cannot*  verify ORCID iDs on behalf of authors.

We look forward to receiving your revised manuscript.

Kind regards,

Samuel Kofi Tchum, Ph.D.

Academic Editor

PLOS One

Journal requirements:

When submitting your revision, we need you to address these additional requirements.

1. Please ensure that your manuscript meets PLOS ONE's style requirements, including those for file naming. The PLOS ONE style templates can be found at

https://journals.plos.org/plosone/s/file?id=wjVg/PLOSOne_formatting_sample_main_body.pdf and

https://journals.plos.org/plosone/s/file?id=ba62/PLOSOne_formatting_sample_title_authors_affiliations.pdf

2. Your ethics statement should only appear in the Methods section of your manuscript. If your ethics statement is written in any section besides the Methods, please move it to the Methods section and delete it from any other section. Please ensure that your ethics statement is included in your manuscript, as the ethics statement entered into the online submission form will not be published alongside your manuscript.

If the reviewer comments include a recommendation to cite specific previously published works, please review and evaluate these publications to determine whether they are relevant and should be cited. There is no requirement to cite these works unless the editor has indicated otherwise.

[Note: HTML markup is below. Please do not edit.]

Reviewers' comments:

Reviewer's Responses to Questions

**Comments to the Author**

1. Is the manuscript technically sound, and do the data support the conclusions?

The manuscript must describe a technically sound piece of scientific research with data that supports the conclusions. Experiments must have been conducted rigorously, with appropriate controls, replication, and sample sizes. The conclusions must be drawn appropriately based on the data presented.

Reviewer #1: Partly

Reviewer #2: Partly

Reviewer #3: Yes

Reviewer #4: Yes

Reviewer #5: No

**********

2. Has the statistical analysis been performed appropriately and rigorously?

Reviewer #1: No

Reviewer #2: Yes

Reviewer #3: Yes

Reviewer #4: Yes

Reviewer #5: No

**********

3. Have the authors made all data underlying the findings in their manuscript fully available?

The PLOS Data policy requires authors to make all data underlying the findings described in their manuscript fully available without restriction, with rare exception (please refer to the Data Availability Statement in the manuscript PDF file). The data should be provided as part of the manuscript or its supporting information, or deposited to a public repository. For example, in addition to summary statistics, the data points behind means, medians and variance measures should be available. If there are restrictions on publicly sharing data—e.g. participant privacy or use of data from a third party—those must be specified.

Reviewer #1: Yes

Reviewer #2: Yes

Reviewer #3: Yes

Reviewer #4: Yes

Reviewer #5: Yes

**********

4. Is the manuscript presented in an intelligible fashion and written in standard English?

PLOS ONE does not copyedit accepted manuscripts, so the language in submitted articles must be clear, correct, and unambiguous. Any typographical or grammatical errors should be corrected at revision, so please note any specific errors here.

Reviewer #1: No

Reviewer #2: Yes

Reviewer #3: Yes

Reviewer #4: Yes

Reviewer #5: No

**********

5. Review Comments to the Author

Please use the space provided to explain your answers to the questions above. You may also include additional comments for the author, including concerns about dual publication, research ethics, or publication ethics. (Please upload your review as an attachment if it exceeds 20,000 characters)

Reviewer #1: Major Comments

• The abstract and discussion sections mentioned performance, sensitivity, and specificity, but these were not described in the methods and results sections. Please describe how the performance was calculated, the definition of True Positives, True Negatives, etc., and include their results.

• Many of the comparisons in the result section were stated without their p-values, which omitted valuable information

• Some of the comparisons in the result section were unclear about their comparator, making it difficult to understand what was being compared and what was the definition “standard” or “correct” classification. It would be best if what was considered as the “correct” classification was described prior, in the methods section.

• The results from spectrophotometry measurements were discussed as often as the results from Biosensor, but the urgency and relation to the study aim was quite unclear.

• For example under the subheading “G6PD activity by Biosensor: comparison between cord blood and follow-up capillary samples”, many of the findings described were based on spectrophotometry measurements. I suggest a separate subheading to describe spectro results to avoid confusion

Comments

• General comments

o Kindly observe the use of correct English grammar throughout the text

o Please spell out abbreviations when they are first used, followed by the abbreviation in brackets. For example, “wild type (WT)”.

• Abstract

o Lines 49-51 “Performance of the point-of-care test as compared to the gold standard spectrophotometry remained excellent at all sampling time-points.”: I find this statement to be misleading because the performance measures of the Biosensor were not reported in the text.

o Lines 51-52 “G6PD activity assessed longitudinally in the same participants decreased over time,”: Only the decrease of median G6PD activity was reported (Table 1 and Table 2); it would be great if the individual decrease in activities was also presented to support this statement. Please also specify that the decrease happens after the D7 measurement, following an increase in H24.

• Methods

o Lines 113-114 “Neonates are classified based on thresholds established before”: Please cite the appropriate reference.

o Lines 114-115 “deficient (≤4.8 IU/gHb), intermediate (4.9-9.9IU/gHb, females only) and normal (≥10.0IU/gHb).”: The classification can be clarified. For example, what will be the status of a male baby with 6 U/g Hb activity?

o Line 169: What's the rationale behind presenting min-max instead of IQR? Also please write minimum and maximum appropriately without abbreviating.

o Lines 169-171 “Correlation between activity in cord blood and at follow-up was assessed using Pearson’s coefficient of correlation.”: I would suggest doing a Bland-Altman analysis as well.

o Line 171: Kindly define what was referred to as “paired samples” prior to this sentence. Did you mean cord blood vs capillary follow-up measurements from the same individual?

• Results

o Lines 205-208: Is there a particular reason the age of the first capillary sample was presented with mean/SD instead of in a similar format with the age of the capillary samples? From Figure S1, the average age of the first capillary sample doesn't seem to be normally distributed.

o Line 222: Spell out CBC when first using any abbreviations

o Line 226 “a new mutation 50G>A”: Please clarify what this mutation position corresponds to (DNA? cDNA?) and which reference sequence was used to establish the position.

o Table 3: Please spell out abbreviations when first used. If there are any abbreviations in Tables or Figures, please spell them in the Table's footnotes or Figure's caption

o Line 235 “(p<0.01)”: Generally, p-values are written with 3 decimal points. If the p-value was between 0.01 and 0.001, please write the exact value.

o Line 236 “G6PD activity in paired samples was significantly higher at 24 hours …”:

• Were you referring to median G6PD activity?

• I’m still unsure about the definition of ‘paired samples’. Were you referring to the capillary samples at 24 hours, and not actually talking about a pair of samples? Please clarify

• If statistical tests comparing the capillary blood with the cord blood per G6PD status category were performed, please include the p-values in Table 1

o Lines 238-239 “Activity was not different in G6PD normal participants analysed by either method”: Kindly add the p-values from the Wilcoxon Signed-Rank Test or paired T-test to support this sentence.

o Lines 246-248 “When comparing status classification, all G6PD deficient participants but one (a deficient heterozygote) would have been correctly identified as deficient by 24-hours and day-7 capillary blood using cord blood thresholds by Biosensor (Figure 2A) and spectrophotometry.”: Please clarify what was referred to as the correct/reference identification here, was it cord blood spectrophotometry? Cord blood Biosensor?

o Figure 2A: Could you please clarify what the asterisk signs in the graph mean? And how are they different from the outlier dots

o Figure 2B: Please add the dotted lines for the thresholds to Fig 2 B

o Lines 250-252 “At 24 hours, there were 6 participants with intermediate activity who would be classified differently if analysis was carried out in capillary blood rather than cord blood …”: But the cord blood was taken during birth and not at 24 hours? I suggest rewording the sentence. Were you referring to the cap blood threshold vs cord blood threshold, rather than talking about the actual samples?

o Paragraph starting at line 246: So in this paragraph, the cord blood measurements by Biosensor and Spectro were treated as the “correct” identification? Is there any biological rationale for this? If not, I would change the term “correctly identified” in this paragraph and stress that this was just a comparison of status identification

o Table 4 and Table 5: Please repeat in the table's footnote what the red highlights mean.

o Line 282: Spell out abbreviation when first use (ICC)

o Line 303 “In particular, significant increase in WBC, RBC count and reticulocyte percentage at <24h of life”: Please state the respective p-value of each increase

o Line 304 “in all G6PD phenotypic groups”: Table S5 need to be stratified by phenotypic groups to support the findings stated in this sentence

• Discussion

o Lines 337-338: Please add the citation using a proper format

o Line 341 “A Second study”: Add citation

o Line 344 “Table S6 shows results …”: Table S6 showed thresholds, not results?

o Lines 348-351: These findings were already described in the Results section

o Lines 353-355: There was no sensitivity or specificity among the findings described in the results section. Either describe these results or refrain from discussing them.

o Lines 358-360 “blood indices are generally similar but tend to be higher in neonatal blood (Greer, Safarulla et al. 2019), particularly for WBC (Scheffer-Mendoza, Espinosa-Padilla et al. 2020)”: How were the results in this study compared to the results in these articles? Do they agree with each other? Are they contradictory?

o Lines 363-365 “increased number of WBC and young reticulocytes would have impacted on total detected activity, especially in G6PD deficient and intermediate samples.”: Please add an appropriate citation

Reviewer #2: The authors present a relatively quick and effective diagnostic PoC test for neonatal G6PD quantitation. The work is succinctly presented and the manuscript itself is well-written. Adding a few comments can help improve the depth of the manuscript:

1. The authors present a good discussion on the correlation between sex and diagnostic threshold. In tables 3-5, presenting the data in correlation with the sex of the neonate can be helpful in drawing more conclusions.

2. How sensitive is the accuracy of G6PD-deficiency classification if the diagnostic thresholds were to be adjusted for (i) different time points, and (ii) cord blood v/s capillary blood? Please comment.

3. The authors should talk more about the reliability of this test in deficient patients with novel and/or rare mutations.

Reviewer #3: - Consider whether the information presented in this paper can be consolidated without compromising your conclusions. The results section, in particular, is quite lengthy, and trimming the wordcount might improve readability.

- Consider whether it may be appropriate to separate your discussion of analysis and results in males vs. females by sex, as the current discussion is somewhat confusing and does not seem to be clearly reflected in the tables.

- Please include units in the tables.

- A discussion about costs of acquiring and using the STANDARD G6PD Biosensor test would be useful when discussing more widespread implementation of this test.

- I would suggest moving the discussion paragraph spanning lines 357-365 earlier in the discussion, as it provides useful context for the rest of the information presented.

Reviewer #4: 1. The primary aim is not consistent to the title. Secondary aim is more suited with the title, therefore should be switched with the primary aim, to better suit the title

2. Please add stronger implication on why "STANDARD-G6PD" tool is chosen to be compared to the gold standard

3. Please add chosen method of statistical analysis in the methods of abstract

4. Related to point (1), conclusion does answer the objectives. However, it mainly answers secondary aim, not the primary one

5. References has not been written in superscript mode, at the end of each word. The reference list at the end should be listen in numbers

Reviewer #5: 1. The data analysis is problematic: the sample sizes are inconsistent across the tables.for example, the numbers of samples reported in Tables 3, 4, and 5 do not match.

2. The clinical issues are unclear, and there are design flaws. This situation more closely resembles the design of a diagnostic trial. Why is it based on the G6PD correlation between peripheral blood and cord blood to prove that the new detection method is more effective?

3.That table also has issues. The standard is a three-column table.

4. The study design lacks a work efficiency curve analysis comparing the experimental group and the gold standard. No cost comparison analysis is provided in the article, which prevents the conclusions from being effectively validated.

**********

6. PLOS authors have the option to publish the peer review history of their article (what does this mean?). If published, this will include your full peer review and any attached files.

If you choose “no”, your identity will remain anonymous but your review may still be made public.

**Do you want your identity to be public for this peer review?**  For information about this choice, including consent withdrawal, please see our Privacy Policy.

Reviewer #1: No

Reviewer #2: No

Reviewer #3: No

Reviewer #4: **Yes:** Pustika Amalia Wahidiyat

Reviewer #5: No

**********

[NOTE: If reviewer comments were submitted as an attachment file, they will be attached to this email and accessible via the submission site. Please log into your account, locate the manuscript record, and check for the action link "View Attachments". If this link does not appear, there are no attachment files.]

To ensure your figures meet our technical requirements, please review our figure guidelines: https://journals.plos.org/plosone/s/figures

You may also use PLOS’s free figure tool, NAAS, to help you prepare publication quality figures: https://journals.plos.org/plosone/s/figures#loc-tools-for-figure-preparation.

NAAS will assess whether your figures meet our technical requirements by comparing each figure against our figure specifications.

Attachment Submitted filename: Review PONE_D_26-14074.docx

# Author comment: Author response to Decision Letter 1

**DOI:** 10.1371/journal.pone.0346837.r002

30 May 2026

Reviewer 1

Major Comments

• The abstract and discussion sections mentioned performance, sensitivity, and specificity, but these were not described in the methods and results sections. Please describe how the performance was calculated, the definition of True Positives, True Negatives, etc., and include their results.

We appreciate the wording of title and abstract might have been confusing but the device has been already fully evaluated before. The title included “performance” meant in the broader sense to include all study aims explained in the abstract and introduction (“The primary aim of the study was to assess equivalency of cord blood and neonatal capillary blood for diagnosis of G6PD deficiency using the quantitative point-of-care “STANDARD G6PDTM” test (SD Biosensor, Korea). Additional secondary aims were to compare the “STANDARD G6PDTM” with gold standard spectrophotometry and to analyse changes in G6PD activity in the first 4 months of life.”)

The abstract does not mention sensitivity and specificity explicitly, but following the reviewers’ comments, we have modified the title and the abstract.

• Many of the comparisons in the result section were stated without their p-values, which omitted valuable information

These have now been added

• Some of the comparisons in the result section were unclear about their comparator, making it difficult to understand what was being compared and what was the definition “standard” or “correct” classification. It would be best if what was considered as the “correct” classification was described prior, in the methods section.

The reference classification used in the manuscript was reported in both the methods (line 110-112) and again in the results (192-194).

• The results from spectrophotometry measurements were discussed as often as the results from Biosensor, but the urgency and relation to the study aim was quite unclear.

The main objective of the study was not to re-evaluate the performance of the device against the spectrophotometry and the study was not powered for this. But since the spectrophotometry was carried out in all samples, we decided to show the results (using Bland-Altman plots) to confirm that performance of the device did not deviate from what already observed in cord blood and children/adult blood. In order to make the manuscript more concise and focused, and following the reviewers’ suggestions, we have decided to move results of spectrophotometric analyses to supplementary material to avoid confusion (see also later).

• For example under the subheading “G6PD activity by Biosensor: comparison between cord blood and follow-up capillary samples”, many of the findings described were based on spectrophotometry measurements. I suggest a separate subheading to describe spectro results to avoid confusion

Thanks for the suggestion. We have revised the result section and moved the results of the spectrophotometer in the supplementary section to avoid confusion

Comments

• General comments

o Kindly observe the use of correct English grammar throughout the text

We have revised the manuscript for English grammar

o Please spell out abbreviations when they are first used, followed by the abbreviation in brackets. For example, “wild type (WT)”.

This was done

• Abstract

o Lines 49-51 “Performance of the point-of-care test as compared to the gold standard spectrophotometry remained excellent at all sampling time-points.”: I find this statement to be misleading because the performance measures of the Biosensor were not reported in the text.

We have re-phrased as “Agreement of G6PD activity of the point-of-care test as compared to the gold standard spectrophotometry remained excellent at all sampling time-points.” These data are reported in Table 6 and in Bland Altman graphs in Figure S9 to S13

o Lines 51-52 “G6PD activity assessed longitudinally in the same participants decreased over time,”: Only the decrease of median G6PD activity was reported (Table 1 and Table 2); it would be great if the individual decrease in activities was also presented to support this statement. Please also specify that the decrease happens after the D7 measurement, following an increase in H24.

As shown and explained in much details later, the activity increases at H24 but then decreases from 1 week to 4 months. Line 51-52 is a general statement in the abstract which reflects the overall (cord blood to 4 month) time course.

In terms of showing individual activities changes over time, we have now added a new Figure (Figure S2) in supplementary file 1.

• Methods

o Lines 113-114 “Neonates are classified based on thresholds established before”: Please cite the appropriate reference.

This was added

o Lines 114-115 “deficient (≤4.8 IU/gHb), intermediate (4.9-9.9IU/gHb, females only) and normal (≥10.0IU/gHb).”: The classification can be clarified. For example, what will be the status of a male baby with 6 U/g Hb activity?

This has been clarified.

o Line 169: What's the rationale behind presenting min-max instead of IQR? Also please write minimum and maximum appropriately without abbreviating.

We have corrected the appropriate spelling of minimum and maximum throughout the manuscript. The rationale for presenting minimum and maximum lays in the use of activity categorizations; we believe that reporting minimum and maximum values recorded at each time point can give a better indication of how spread results were and how many were outside the pre-established cut-offs.

o Lines 169-171 “Correlation between activity in cord blood and at follow-up was assessed using Pearson’s coefficient of correlation.”: I would suggest doing a Bland-Altman analysis as well.

We have to disagree with the reviewer on this point as Bland-Altman analysis is not appropriate for this type of analysis. We have used it instead when comparing results from different tests carried out in the same sample.

o Line 171: Kindly define what was referred to as “paired samples” prior to this sentence. Did you mean cord blood vs capillary follow-up measurements from the same individual?

Yes, paired samples were samples collected over time in the same individual.

• Results

o Lines 205-208: Is there a particular reason the age of the first capillary sample was presented with mean/SD instead of in a similar format with the age of the capillary samples? From Figure S1, the average age of the first capillary sample doesn't seem to be normally distributed.

As highlighted by the reviewer, mean/SD was not the correct way to show the age of first capillary sample and it has now been corrected.

The age is not normally distributed because the indication was to collect the sample within the first 24h of life, as close as possible to the 24h mark; considerations about reducing the number of needle sticks in neonates who needed blood for clinical reasons and avoiding nightly collections were also taken into account, therefore some of the blood samples were collected much earlier than 24 hours.

o Line 222: Spell out CBC when first using any abbreviations

This was done

o Line 226 “a new mutation 50G>A”: Please clarify what this mutation position corresponds to (DNA? cDNA?) and which reference sequence was used to establish the position.

This is the position of the coding DNA; reference sequence is ENSG00000160211 (transcript G6PD-006). This was added to the manuscript

o Table 3: Please spell out abbreviations when first used. If there are any abbreviations in Tables or Figures, please spell them in the Table's footnotes or Figure's caption

This was done

o Line 235 “(p<0.01)”: Generally, p-values are written with 3 decimal points. If the p-value was between 0.01 and 0.001, please write the exact value.

This was done

o Line 236 “G6PD activity in paired samples was significantly higher at 24 hours …”:

§ Were you referring to median G6PD activity?

§ I’m still unsure about the definition of ‘paired samples’. Were you referring to the capillary samples at 24 hours, and not actually talking about a pair of samples? Please clarify

§ If statistical tests comparing the capillary blood with the cord blood per G6PD status category were performed, please include the p-values in Table 1

This was included in the text at Line 232-238 in the revised version

o Lines 238-239 “Activity was not different in G6PD normal participants analysed by either method”: Kindly add the p-values from the Wilcoxon Signed-Rank Test or paired T-test to support this sentence.

P-values from the Wilcoxon Signed-Rank Test were added for all comparisons

o Lines 246-248 “When comparing status classification, all G6PD deficient participants but one (a deficient heterozygote) would have been correctly identified as deficient by 24-hours and day-7 capillary blood using cord blood thresholds by Biosensor (Figure 2A) and spectrophotometry.”: Please clarify what was referred to as the correct/reference identification here, was it cord blood spectrophotometry? Cord blood Biosensor?

This referred to only Biosensor, the words “and spectrophotometry” were a mistake (since the data for spectrophotometry were reported later). We have corrected the text and moved the spectrophotometry section to the supplementary file 2.

o Figure 2A: Could you please clarify what the asterisk signs in the graph mean? And how are they different from the outlier dots

In box-plots, SPSS used asterisk to indicate extreme outliers (>3 time the height of the box) as compared to circles which indicate outliers (values that do no fall in the inner fences). This has been added to the caption.

o Figure 2B: Please add the dotted lines for the thresholds to Fig 2 B

There are no dotted lines in this Figure because G6PD genotypes in females are not expected to be associated with any specific activity range

o Lines 250-252 “At 24 hours, there were 6 participants with intermediate activity who would be classified differently if analysis was carried out in capillary blood rather than cord blood …”: But the cord blood was taken during birth and not at 24 hours? I suggest rewording the sentence. Were you referring to the cap blood threshold vs cord blood threshold, rather than talking about the actual samples?

This indicates that using the cord-blood derived thresholds for classification of activity in capillary blood collected at 24h would result in misclassification of 6 participants. This has been re-phrased for clarity.

The way this analysis was carried out is explained in the methods.

o Paragraph starting at line 246: So in this paragraph, the cord blood measurements by Biosensor and Spectro were treated as the “correct” identification? Is there any biological rationale for this? If not, I would change the term “correctly identified” in this paragraph and stress that this was just a comparison of status identification

The primary aim of the study was to see whether thresholds already validated in cord blood could be used in capillary blood collected during the first week of life. Therefore, the classification of participants based on the cord blood is considered the correct identification.

Practically, this has large impact on the way G6PD activity can be tested in neonates and high risk of jaundice be identified.

o Table 4 and Table 5: Please repeat in the table's footnote what the red highlights mean.

This was done

o Line 282: Spell out abbreviation when first use (ICC)

This was done

o Line 303 “In particular, significant increase in WBC, RBC count and reticulocyte percentage at <24h of life”: Please state the respective p-value of each increase

Added

o Line 304 “in all G6PD phenotypic groups”: Table S5 need to be stratified by phenotypic groups to support the findings stated in this sentence

There is no difference by phenotypic groups as clearly visible in Figure S5 A to D; the comparison is between cord blood and 24h sample, not among different phenotypic group. All comparisons are statistically significant at the p<0.001 level aside from reticulocytes in G6PD normal where the p is 0.003

• Discussion

o Lines 337-338: Please add the citation using a proper format

This has been corrected

o Line 341 “A Second study”: Add citation

The reference was added

o Line 344 “Table S6 shows results …”: Table S6 showed thresholds, not results?

We have modified this sentence

o Lines 348-351: These findings were already described in the Results section

Yes this findings were already described in results and they were redundant. We have delete them here.

o Lines 353-355: There was no sensitivity or specificity among the findings described in the results section. Either describe these results or refrain from discussing them.

We think a consideration about the lack of sensitivity or specificity can be made based on available data even without a formalized sensitivity and specificity analysis.

o Lines 358-360 “blood indices are generally similar but tend to be higher in neonatal blood (Greer, Safarulla et al. 2019), particularly for WBC (Scheffer-Mendoza, Espinosa-Padilla et al. 2020)”: How were the results in this study compared to the results in these articles? Do they agree with each other? Are they contradictory?

They all go on the same direction. We have re-phrased for clarity.

o Lines 363-365 “increased number of WBC and young reticulocytes would have impacted on total detected activity, especially in G6PD deficient and intermediate samples.”: Please add an appropriate citation

Two citations were added.

Reviewer 2

The authors present a relatively quick and effective diagnostic PoC test for neonatal G6PD quantitation. The work is succinctly presented and the manuscript itself is well-written. Adding a few comments can help improve the depth of the manuscript:

1. The authors present a good discussion on the correlation between sex and diagnostic threshold. In tables 3-5, presenting the data in correlation with the sex of the neonate can be helpful in drawing more conclusions.

This is a good suggestion and usually we would show data separately for males and females. In this case we are dealing mostly with G6PD phenotypes and the associated risk of jaundice which is directly linked to the phenotype (regardless of the sex). For this reason, we feel that following a phenotypic categorization is more appropriate.

2. How sensitive is the accuracy of G6PD-deficiency classification if the diagnostic thresholds were to be adjusted for (i) different time points, and (ii) cord blood v/s capillary blood? Please comment.

While such an analysis might be intriguing, the study was not designed to assess sensitivity of different diagnostic thresholds at different time points so, unfortunately we cannot answer this question.

3. The authors should talk more about the reliability of this test in deficient patients with novel and/or rare mutations.

In this study we only found a novel mutation in a heterozygote participant so we do not know what level of residual activity is associated to the mutation in hemizygote state. Since the test assesses G6PD activity and not genotype, we would expect the reliability be the same across genotypes.

Reviewer 3

Consider whether the information presented in this paper can be consolidated without compromising your conclusions. The results section, in particular, is quite lengthy, and trimming the wordcount might improve readability.

We agree with the reviewer and have decided to move the comparisons of activity by spectrophotometry to supplementary file 2.

- Consider whether it may be appropriate to separate your discussion of analysis and results in males vs. females by sex, as the current discussion is somewhat confusing and does not seem to be clearly reflected in the tables.

This is a good suggestion but since we are dealing mostly with phenotypes, and risk of jaundice is directly linked to phenotype, we feel that following a phenotypic categorization is more appropriate.

- Please include units in the tables.

Where missing, units have been included in the titles or Tables in both main Table and supplementary files

- A discussion about costs of acquiring and using the STANDARD G6PD Biosensor test would be useful when discussing more widespread implementation of this test.

A discussion about the cost of using the STAN

Attachment Submitted filename: Responses_to_reviewers_final.docx

# Peer review: Decision Letter 1

**DOI:** 10.1371/journal.pone.0346837.r003

**Authors:** Samuel Tchum, Samuel Tchum

24 Jun 2026

Use of the “STANDARD G6PDTM” quantitative point-of-care test in neonates and infants

PONE-D-26-14074R1

Dear Dr. Bancone,

We’re pleased to inform you that your manuscript has been judged scientifically suitable for publication and will be formally accepted for publication once it meets all outstanding technical requirements.

Within one week, you’ll receive an e-mail detailing the required amendments. When these have been addressed, you’ll receive a formal acceptance letter and your manuscript will be scheduled for publication.

An invoice will be generated when your article is formally accepted. Please note, if your institution has a publishing partnership with PLOS and your article meets the relevant criteria, all or part of your publication costs will be covered. Please make sure your user information is up-to-date by logging into Editorial Manager at Editorial Manager® and clicking the ‘Update My Information' link at the top of the page. For questions related to billing, please contact billing support.

If your institution or institutions have a press office, please notify them about your upcoming paper to help maximize its impact. If they’ll be preparing press materials, please inform our press team as soon as possible -- no later than 48 hours after receiving the formal acceptance. Your manuscript will remain under strict press embargo until 2 pm Eastern Time on the date of publication. For more information, please contact onepress@plos.org.

Kind regards,

Samuel Kofi Tchum, Ph.D.

Academic Editor

PLOS One

Additional Editor Comments (optional):

Reviewers' comments:

Reviewer's Responses to Questions

**Comments to the Author**

1. If the authors have adequately addressed your comments raised in a previous round of review and you feel that this manuscript is now acceptable for publication, you may indicate that here to bypass the “Comments to the Author” section, enter your conflict of interest statement in the “Confidential to Editor” section, and submit your "Accept" recommendation.

Reviewer #1: All comments have been addressed

Reviewer #2: (No Response)

Reviewer #5: (No Response)

**********

2. Is the manuscript technically sound, and do the data support the conclusions?

The manuscript must describe a technically sound piece of scientific research with data that supports the conclusions. Experiments must have been conducted rigorously, with appropriate controls, replication, and sample sizes. The conclusions must be drawn appropriately based on the data presented.

Reviewer #1: Yes

Reviewer #2: Partly

Reviewer #5: Yes

**********

3. Has the statistical analysis been performed appropriately and rigorously?

Reviewer #1: Yes

Reviewer #2: Yes

Reviewer #5: No

**********

4. Have the authors made all data underlying the findings in their manuscript fully available?

The PLOS Data policy requires authors to make all data underlying the findings described in their manuscript fully available without restriction, with rare exception (please refer to the Data Availability Statement in the manuscript PDF file). The data should be provided as part of the manuscript or its supporting information, or deposited to a public repository. For example, in addition to summary statistics, the data points behind means, medians and variance measures should be available. If there are restrictions on publicly sharing data—e.g. participant privacy or use of data from a third party—those must be specified.

Reviewer #1: Yes

Reviewer #2: Yes

Reviewer #5: Yes

**********

5. Is the manuscript presented in an intelligible fashion and written in standard English?

PLOS ONE does not copyedit accepted manuscripts, so the language in submitted articles must be clear, correct, and unambiguous. Any typographical or grammatical errors should be corrected at revision, so please note any specific errors here.

Reviewer #1: Yes

Reviewer #2: Yes

Reviewer #5: Yes

**********

6. Review Comments to the Author

Please use the space provided to explain your answers to the questions above. You may also include additional comments for the author, including concerns about dual publication, research ethics, or publication ethics. (Please upload your review as an attachment if it exceeds 20,000 characters)

Reviewer #1: All comments have been addressed satisfactorily. This includes major and minor suggestions from all reviewers.

Reviewer #2: Although the manuscript succinctly presents a unique perspective on the POC, quite a bit remains to be reviewed for the test to be robustly validated.

Reviewer #5: Compared to the first draft, this version has a much better logical structure in both the research purpose and the subsequent discussion sections. I still suggest using a three-line table format for the statistical table, which will help maintain the uniformity of the magazine. I can attach the template of the three-line table by e-mail.

**********

7. PLOS authors have the option to publish the peer review history of their article (what does this mean?). If published, this will include your full peer review and any attached files.

If you choose “no”, your identity will remain anonymous but your review may still be made public.

**Do you want your identity to be public for this peer review?**  For information about this choice, including consent withdrawal, please see our Privacy Policy.

Reviewer #1: No

Reviewer #2: No

Reviewer #5: No

**********

Attachment Submitted filename: three-line table model.png

# Editor report: Acceptance letter

**DOI:** 10.1371/journal.pone.0346837.r004

**Authors:** Samuel Tchum, Samuel Tchum

PONE-D-26-14074R1

PLOS One

Dear Dr. Bancone,

I'm pleased to inform you that your manuscript has been deemed suitable for publication in PLOS One. Congratulations! Your manuscript is now being handed over to our production team.

At this stage, our production department will prepare your paper for publication. This includes ensuring the following:

* All references, tables, and figures are properly cited

* All relevant supporting information is included in the manuscript submission,

* There are no issues that prevent the paper from being properly typeset

You will receive further instructions from the production team, including instructions on how to review your proof when it is ready. Please keep in mind that we are working through a large volume of accepted articles, so please give us a few days to review your paper and let you know the next and final steps.

Lastly, if your institution or institutions have a press office, please let them know about your upcoming paper now to help maximize its impact. If they'll be preparing press materials, please inform our press team within the next 48 hours. Your manuscript will remain under strict press embargo until 2 pm Eastern Time on the date of publication. For more information, please contact onepress@plos.org.

You will receive an invoice from PLOS for your publication fee after your manuscript has reached the completed accept phase. If you receive an email requesting payment before acceptance or for any other service, this may be a phishing scheme. Learn how to identify phishing emails and protect your accounts at https://explore.plos.org/phishing.

If we can help with anything else, please email us at customercare@plos.org.

Thank you for submitting your work to PLOS ONE and supporting open access.

Kind regards,

PLOS ONE Editorial Office Staff

on behalf of

Dr Samuel Kofi Tchum

Academic Editor

PLOS One
